# Pride and confidence at work: potential predictors of occupational health in a hospital setting

**DOI:** 10.1186/1471-2458-5-92

**Published:** 2005-09-01

**Authors:** Kerstin Nilsson, Anna Hertting, Inga-Lill Petterson, Töres Theorell

**Affiliations:** 1 School of Life Sciences, University of Skövde, Skövde, Sweden; 2 National Institute for Psychosocial Medicine (IPM), Stockholm, Sweden; 3 Department of Public Health Sciences and Department of Occupational and Environmental Health, Stockholm, Sweden

## Abstract

**Background:**

This study focuses on determinants of a healthy work environment in two departments in a Swedish university hospital. The study is based on previously conducted longitudinal studies at the hospital (1994–2001), concerning working conditions and health outcomes among health care personnel in conjunction with downsizing processes. Overall, there was a general negative trend in relation to mental health, as well as long-term sick leave during the study period. The two departments chosen for the current study differed from the general hospital trend in that they showed stable health development. The aim of the study was to identify and analyse experiential determinants of healthy working conditions.

**Methods:**

Thematic open-ended interviews were carried out with seventeen managers and key informants, representing different groups of co-workers in the two departments. The interviews were transcribed verbatim and an inductive content analysis was made.

**Results:**

In the two studied departments the respondents perceived that it was advantageous to belong to a small department, and to work in cooperation-oriented care. The management approaches described by both managers and co-workers could be interpreted as transformational, due to a strain of visionary, delegating, motivating, confirmative, supportive attitudes and a strongly expressed solution-oriented attitude. The daily work included integrated learning activities. The existing organisational conditions, approaches and attitudes promoted tendencies towards a work climate characterised by trust, team spirit and professionalism. In the description of the themes organisational conditions, approaches and climate, two core determinants, work-pride and confidence, for healthy working conditions were interpreted. Our core determinants augment the well-established concepts: manageability, comprehensiveness and meaningfulness. These favourable conditions seem to function as a buffer against the general negative effects of downsizing observed elsewhere in the hospital, and in the literature.

**Conclusion:**

Research illuminating health-promoting aspects is rather unusual. This study could be seen as explorative. The themes and core dimensions we found could be used as a basis for further intervention studies in similar health-care settings. The result could also be used in future health promotion studies in larger populations. One of the first steps in such a strategy is to formulate relevant questions, and we consider that this study contributes to this.

## Background

In this study we have focused on potential determinants of healthy working conditions in two departments in a Swedish university hospital. In previously conducted longitudinal studies at the present hospital (1994–2001), Hertting and colleagues studied working conditions and health outcomes among health care personnel in conjunction with downsizing processes during the 1990s. This downsizing period was characterised by personnel reductions; the hospital staff was reduced by 22% (1000 persons) between 1995–1997, and 10% were relocated to other departments. Other structural changes during the study period were mergers of departments and outsourcing of service units, along with continuous demands for cutting costs [1-5].

Biological stress markers, measured on 31 female medical secretaries, registered nurses and assistant nurses, indicated that protective and anabolic functions had suffered, from the adjustment phase (1997) to the reconstruction in 1998. The continuing adaptation process indicated increasing difficulty for the women to mobilise energy [1]. Repeated interviews (1997, 1998, 2000 and 2001) with the same respondents confirmed these difficulties [2-4].

A third study, based on a questionnaire and hospital register data (1994–2001), found evidence for negative trends in mental health and in long-term sick leave at the hospital, corresponding to negative trends in working conditions [5]. In international research, increasing work demands and reduced control [6], combined with job insecurity and loss of trust [7], were identified during periods of downsizing. Further, downsizing has been associated with subsequent high rates of sickness absence [8].

Petterson and her colleagues [5] also found that increasing demands to work hard, conflicting demands, and increasing lack of time to plan work, were strongly associated with overall deteriorating health. It was not possible to identify health-promoting departments in terms of positive health development. However, compared with the generally negative hospital trend, four of the 24 departments studied might be labelled more 'health-promoting', since they showed stable trends both in mental health and in short- and long-term sick-leave rate. Two of these departments were selected for this current interview study.

### Health promotion at the workplace

A health-promoting hospital has been considered to be a 'healthy organisation' in the sense of having an effective relation-oriented management and active staff participation [9], which means a workplace where people are able to produce, serve, grow and be evaluated [10]. Accordingly, in the context of working life, health promotion should be a matter of supporting peoples' resources by enhancing job control, encouraging social support, networks and relations at the workplace, and providing a meaningful job.

Health promotion is often based on the salutogenic model [11], where health is seen as being created by a sense of coherence, including comprehensibility, meaningfulness and manageability. When applied to working life, comprehensibility implies that work changes have to be predictable. Work assignments must be meaningful in the sense of being perceived as socially rewarding and stimulating. Manageability means access to the necessary resources for handling work pressure, which includes own ability as well as material and supportive resources provided by the environment. Health promotion at the workplace could be a question of enhancing such resources by strengthening the individual's internal control and ability to act, i.e. room for manoeuvre [12].

Ever since the demand-control model was introduced [13], increased decision latitude has been perceived as an important element in health promotion in the workplace. In a longitudinal study an improved relationship between decision latitude and psychological demands has been shown to be related to a rising serum concentration of testosterone, which is an index of increasing regenerative bodily activity [14]. In different controlled experiments it has been shown that efforts to improve decision latitude for employees are associated with improvements in morning serum cortisol [15], decreased staff turnover and decreased sick leave [16].

Health outcomes could be predicted, as there is a correlation between positive self-rated health and a lower risk of future sickness and death [17]. Research has also found that satisfaction with one's place of work and profession are important predictors for positive good health [18]. In a longitudinal participatory worksite intervention, job control was recognised as the mediator for increased mental health, less sick leave and a higher level of self-rated performance [19]. In another extensive longitudinal study in a health-care setting it was found that workmates or managers freezing out employees, and employees' experiences of negative and threatening changes at the workplace are of importance for long-term sick leave [20].

In a survey study, Noblet [21] confirmed that job control and social support at work had a health-promoting value. Job stressors accounted for as much as 25% of explained variance in mental health. The factor most strongly associated with mental health and job satisfaction was time to do the job as well as you would like. Time-related pressure was strongly associated with job strain.

Skill variety, autonomy, social contact and learning were confirmed as determinants of intrinsic work motivation in a study of specific work-setting determinants for contributing to the motivation and good health of nurses. These factors made their work challenging and meaningful. Emotional exhaustion can be reduced if attention is paid to workload [22].

### Leadership and employee health

Transformational leadership contains elements of charismatic leadership, inspirational motivation, intellectual stimulation and individual consideration [23]. Bass and Avolio found that leaders with a transformational style are capable of empowering employees to stretch their limits, and of being innovative to create solutions [24]. Empowering management could contribute to extended decision latitude, as power in the organisation transfers to co-workers [25]. A workplace environment characterised by such empowerment strategies can improve productivity as well as cohesion and job satisfaction for the employees [26,27]. The relation between health outcome and job satisfaction has been confirmed [28], and job satisfaction among nursing staff has been found to be positively correlated with transformational leadership style [29].

The relationship between organisational efficiency and transformational leadership style was confirmed by Marks-Moran [30]. However, transformational leadership was found to be most efficient when management by team is a reality and the organisation is development-oriented [31]. Bass [23] concluded that transformational leaders are more successful, in terms of efficiency, than leaders using a transactional style, which includes management-by-expectation, and contingent reinforcement and rewards. In a study among Belgian head nurses, fewer work stressors and lower levels of emotional exhaustion were found among staff when a transformational leadership style was used [32]. However, in a study of Swedish health centres, Bernin and Theorell were not able to confirm any impact of leadership style on subordinates' health [33]. From the review of research in the field of management, there are studies supporting the view that leadership is one factor that influences job satisfaction. However, there are different and inconclusive findings concerning leadership style and its impact on employees' health.

In the present study the purpose was to understand how employees succeeded in retaining their health and going beyond the stable (positive) health trends in the two selected departments in a period of turbulence [5]. By focusing on experienced health determinants in management at department levels, we expect to acquire knowledge about health-promotive factors for employees working in health care organisations. In this study we identify management as a phenomenon where both managers and co-workers participate.

The aim of the study was to identify and analyse experiential determinants of healthy working conditions from the perspectives of hospital managers and staff in key positions.

## Methods

Based on previous studies of this particular hospital and on the purpose of the study, thematic open-ended interviews were chosen as a method for data collection. The outcomes of such interviews depend on the quality of interaction between the respondent and the interviewer, but also upon culturally hidden assumptions about understandings of experiences, feelings and intentions [34]. The interview is a face-to-face meeting, where the researcher attempts to understand the respondent's perspective and experiences. This makes the interviews more like a conversation than an interview with scope simply for asking and responding [35]. It is important to mention that it is the outcome of the interviews that is analysed and not the interaction [36].

### Participants and data collection

Interviews with 17 persons were conducted by the first author (KN), in two departments with stable health trends between 1994 and 2001. These particular departments were selected as they had all-round health care, such as nursing wards, assessment units, as well as care and treatment wards for outpatients. One of the departments specialised in neurological care with the district as catchment area, and the other in gynaecological ontological care with an extended region as its catchment area. The respondents were managers and selected key persons among the co-workers, including all managers, both on department and ward level, and co-workers including physicians with medical responsibility, staff with special care responsibility and a trade union representative (see Table 1). Twelve of the interview persons were women and five were men. All of them were middle-aged, and all had experience from the same department during the whole study period (1994–2001).

**Table 1:** Participants

**Participants' function (profession)**	**Number**
Head of department (physician)	2
Ward sister (nurse)	4
Assistant ward sister (nurse)	2
Medically responsible (physician)	3
Responsible for medical secretariat (medical secretary)	2
Responsible for occupational therapy (occupational therapist)	1
Responsible for cancer nursing (nurse)	2
Trade union representatives (assistant nurse)	1

**Total**	**17**

The head**s **of the department**s**, in agreement with the research team, selected the interviewees, who were chosen to reflect varied experiences of working conditions, the leadership in the organisation and professional tasks. All interviews lasted about one hour and were carried out in the autumn of 2003. The thematic interview guide included the following themes: organising the work, handling organisational changes, taking advantage of the co-workers' resources, and the meaning of healthy work. The thematic interviews were unstructured, in accordance with Silverman [34]. This implies that each theme was discussed in every interview, but in different sequences depending on how the interview developed. The interviewer represented the field of nursing research, had many years of experience as a teacher in nursing education, and was trained in interviewing.

### Data analysis

All interviews were transcribed verbatim and included about 170 pages in all of single-spaced text. An inductive content analysis based on a thematic coding of the text was made in order to obtain meaning and understanding [34]. Interviews with respondents from the two departments were analysed together, as the phenomenon of interest was to identify potential contributing factors to stable health. To gain a holistic depiction of the material, all protocols were read in their entirety by the first author (KN). Keeping the aim of the study in mind, words and sentences were marked in the text. Thereafter followed a process where words and sentences (with related segments of interview texts) were brought together in terms of content. These groups were given preliminary names. In order to validate the analysis, the second author (AH) participated as a co-analyser and examined the themes (found in the first analysis by KN) in relation to the material as a whole. The two analysers then compared each other's analyses and the analysing process lasted until agreement was reached. During this comparative procedure the groups were reduced in number and expanded in content to finally form three themes with accompanying sub-themes reported in the results.

### Methodological considerations

Prerequisites for attaining the respondents' descriptions were supported by the interviewers having no previous experience of these departments. Openness and a friendly atmosphere characterised the interview situation, which made it easier for the interviewees to say what they wanted to say in the present situation [34].

In the analysis phase there is always a risk that preconceptions about the studied field of research could influence the process. Preconceptions can be an advantage, as an intimate knowledge of the field facilitates discovery of nuances in the interviewees' statements. But, on the other hand, there is a risk that a field that is too well-known can blind the researcher, who may thereby miss new angles of incidences on the studied phenomenon. To minimise these methodological pitfalls we reflected on this problem together and tried to be aware of our preconceptions in all research phases [34].

In this study we used a co-analyser, which also demands awareness during the analyses. Having a co-analyser could be an advantage, as two people are always able to discover more than one. But it is necessary to point out that there is a risk that the analysers strive for consensus, which in its turn could reduce variation in the content of the themes [34].

In order to validate our tentative findings we gave feedback to the respondents, to receive their reactions [34]. Hence, the findings were discussed in managerial group meetings, resulting in minor modifications. Good agreement supported high face validity.

### Ethical considerations

This study followed the Humanistic-Social Research Council Ethics Rules [37]. Due to ethical considerations, special emphasis was placed on informing the participants about the study, obtaining their consent and treating their statements confidentially. The interviewees have neither before, nor during, nor after the interview occasions refused to participate. The quotations in the results are used to exemplify the statements of the individual respondents and are marked with a figure.

## Results

The analyses resulted in three themes with accompanying sub-themes (see Table 2), indicating a work process emanating from the organisational conditions at the departments; as well as the development of organisational approaches, attitudes and activities that were favourable to the creation of a climate characterised by trust, team spirit and professionalism.

**Table 2:** Themes and sub-themes in the result

**Theme**	**Sub-theme**
Organisational conditions	- The benefits of a small department
	- The benefits of the character of care
Organisational approaches	- Leadership and followership attitudes
	- Learning activities
	- Supportive activities
Organisational climate	- Trust
	- Team spirit
	- Professionalism

### Organisational conditions

#### The benefits of a small department

Approximately 60 people are employed in each department, which the respondents consider to be small departments, with regard to both the size of the staff and the number of beds and outpatient capacity. According to the interviewees, this means that the staff get to know each other and are seen by both managers and each other. Accordingly, distance is reduced between co-workers, as well as between subordinates and employees in different managerial positions, i.e. within the hierarchies.

It's so easy to have an overview of everything. It's much worse in a big department with several hundred employees. There are so few of us that we can all fit into a bus. (8)

The fact that the departments are small, facilitates vertical and horizontal communication between occupational functions and levels, and is judged by the respondents to be very good. Regular meetings are held between managers at different levels, in order to draw up guidelines and to discuss solutions to any problems that may arise.

#### The benefits of the nature of care provided

Another important factor is the nature of the care and treatment given to patients at the departments. Work in the departments is cooperation-oriented. Investigation, diagnosis, treatment and rehabilitation demand the involvement of several professional groups. In many cases the doctor cannot make a medical decision without having heard for example how the assistant nurse or the occupational therapist perceives the patient's functional ability. Planning rounds are mentioned as examples of activities that involve several categories of personnel. In one department the sense of belonging is created in the need for working together in caring for patients.

It's a question of teamwork. You don't work as a doctor in isolation. You've got to have the whole team, all the personnel categories; otherwise the care won't be good. (14)

The sense of belonging can also be strengthened by making comparisons with similar departments at other hospitals. This could be exemplified by a statement from a respondent from one of the departments where highly specialised skill has been developed.

The speciality that we have is a very narrow field and is rather unique, and of course it feels good to be doing something exclusive. (8)

### Organisational approaches

#### Leadership and followership attitudes

The respondents indicate that it is important that the heads of department work for development, for example strengthening and preserving their departments' specialities, particularly in times of cuts and rationalisation.

Our boss has been strong and has shown that he knows what he's talking about, standing up to them, showing that it works and that it's going to work/.../ My head of department is really good at having something up his sleeve that might be useful for the next round of cuts. (16)

The value of a clear division of responsibility and authority between levels and individuals is a recurrent theme in the interviews, and permeates all parts of the organisation. The group of assistant nurses who remained after the cutbacks of the 90s are valued and defended, and their competence is emphasised by for example delegating to them work tasks that are normally those of the registered nurses. The intention is that all personnel should use their competence to take responsibility for and influence their work.

I think it's important that you give the staff quite a lot of freedom, that they feel that they can use their competence, that I trust them. (7)

At the same time the managers feel that there is a risk that people who need clear guidelines become frustrated when they are given freedom. For this reason, they say, it is necessary to have a balance between freedom and control.

Many employees might think that it's a little bit frustrating with too much freedom, as they want rather clear guidelines. (7)

The informants describe a leadership characterised by inspiration and support towards the co-workers. The management encourages the co-workers to talk to each other about difficult meetings with patients, but it is the co-workers' responsibility to use all available opportunities for support. The co-workers say that they have the support of the management, because they are allowed not to be efficient every single minute; they are given time for reflection.

If we feel that it's hard to go in to a patient we must be able to say so. Then two of us go in instead, or someone else goes in. After all, we've got patients with serious illnesses, young people with brain tumours, and that can be tough to deal with. (9)

In terms of financial cuts and savings, the staff in the two present departments have experienced the same demands as those of other departments at the hospital. They have grappled with cultural clashes when wards have been merged and have had to solve different conflicts between personnel. In this respect these departments are no different from others. What characterises them is rather that managers and co-workers are prepared to solve difficulties that arise themselves. When they have not been able to solve the problems themselves, they have sought help from external experts. Now and then they have been forced to make organisational changes in order to deal with conflicts that have arisen at the departments.

The co-workers are willing to do their bit to help out and cover the staff vacancies that arise. According to the respondents there is a risk that they take on too much extra work out of loyalty. The heads of departments try not to overwork the staff, but do not always succeed. It is easy to exceed the limit for what is a reasonable workload when, according to the heads of departments, the co-workers are willing and interested in the work and readily take on extra work tasks. The heads of department endeavour to give the staff the opportunity to recover their strength between their work shifts. The following excerpt illustrates the reciprocity in the leader – co-worker relationship.

Being willing to help, covering for each other, helping each other, and this knowledge of each other and personal chemistry that's sort of part of the atmosphere here.... I think all that has been really good for us when we've been under stress. (13)

#### Learning activities

Managers encourage the co-workers both to use and deepen their competence, for example by encouraging them to participate in groups which develop new knowledge within a particular area, such as care of brain tumour patients or cytotoxic therapy. These interest groups are responsible for getting information and passing it on to their colleagues.

This multidisciplinary way of working, with everyone focussing on a particular complex of problems, has been rather successful. (13)

The transfer of knowledge has a social value and takes place within and between professional groups. Various opportunities are used for the transfer of knowledge, for example during a break, when a doctor talks about a case: "patient of the week". Another forum where transfer of knowledge takes place is planning rounds, which do not begin until all the professional groups are present. The following excerpt illustrates the importance of everyone's involvement:

We've got to work together with focus on the patient; physiotherapists, occupational therapists, and especially the assistant nurses are there and have their say, because they're the ones who really see the patient. (15)

No matter which professional group or department the respondents represent, the fact that the staff help and learn from each other is a recurrent theme in their statements. There is an open climate, which makes it possible to ask anybody at any time about anything.

They [nurses] really get a lot of help when they're new from those who've been there a while, and of course that's the kind of culture we want to keep. (15)

Apart from these efforts to deepen and use the competence of the co-workers, external networks have been developed among all the occupational groups. These networks are said to contribute to learning and to the development of self-confidence, in that knowledge and skills are acknowledged and anchored outside their own department. Physicians participate in several networks, both nationally and internationally. As far as registered nurses are concerned, it is above all a question of national contacts; something that the assistant nurses have made tentative attempts to establish, while the medical secretaries mainly work in local networks.

#### Supportive activities

Resources, above all staff resources, are insufficient at both departments, in the same way as at other workplaces. At the same time, the staff at these departments work with patients who are seriously and/or chronically ill. According to the respondents, this means that the staff can get into conflict situations. For this reason routines have been developed, where doctors, registered nurses and assistant nurses make joint decisions about what is to be prioritised for the individual patient if the resources are insufficient. In this way the responsibility for prioritising is removed from the individual caregiver.

We have a written list of priorities. Some days it must be OK that the patients don't get up. We have to set our sights at such a level that the staff can manage, but they shouldn't have to make that decision themselves. (1)

Care of patients who are seriously ill puts a strain on the staff. A situation that is mentioned by several interviewees is the care of patients with incurable diseases, and where the staff can identify themselves with the patient's situation. Apart from being open to getting support from one another in their everyday work there are scheduled routines so that the staff can discuss their experiences, depending on their various needs, with both workmates and other professional actors, e.g. social workers with special training in counselling.

If a patient is in a lot of pain and gets no relief for this pain, no matter what you do, this can lead to serious anxiety in the patient – and of course very often this really gets to you/.../ I think that talking about the care situations we've experienced as difficult makes us understand each other better. (17)

Apart from support in their work there are social activities for staff, both in their leisure time and during working hours, e.g. walks, keep fit classes etc. But according to the respondents, health-promoting activities also include that little gesture of caring about and noticing the co-workers, and of creating a good atmosphere. One of the heads of ward says that such measures to promote well-being are perceived by the staff to mean that the head is involved:

I've been given credit for being a head of ward who cares and is involved. I mean they might say 'We know you're always there for us' (16)

At one of the departments, there has been a tradition right from the start, more than 10 years ago, of going off on a study trip every year, and this is something that all of those who were interviewed at that department valued very highly. The trips are considered to have contributed to a good atmosphere, partly because the staff had got to know each other in a different context, and partly because they had come into contact with other workplaces, and the unique collective knowledge and skills of their department had been acknowledged.

Then we all go on a so-called department trip once a year so that we can be together like 24 hours a day in another environment. I think that's really valuable/.../ and then we usually think that we're really well off compared with other hospitals abroad. (11)

### Organisational climate

#### Trust

In the interviews there is an expression of trust for each other; a trust that the respondents feel depends on the fact that they have confidence in each other's knowledge and skills. Every individual is trusted to carry out her/his duties. This confidence exists between leaders and co-workers, just as it exists between and within different professional groups.

Everyone is tolerant; we don't check up on each other that this and that have been done. We all have our own roles, everyone has their own thing and we know who does what. (16)

The sense of security that has developed makes it easier to be able to deal with difficulties and with individual shortcomings, but it is also the basis for enjoying one's work. Trust, security and the joy of working together means that people are happy at work and there is a good team spirit. This means that they perceive themselves to be equipped to deal with the ups and downs in the burden of health care and staff shortages: situations that can make the small department vulnerable.

#### Team spirit

At the same time as the team spirit is a distinguishing feature of the departments, many of the work tasks are independent ones. A characteristic feature of the studied departments is that cooperation between all professional groups is well developed. Which groups work together depends on the kind of work and the needs of the patients.

Cooperation between different groups is a very characteristic feature of our everyday work in the ward ... we're very dependent on each other, in fact everything is centred on the patient. (5)

Teamwork strengthens the feeling of participation. In the same way, the fact that the levels in the hierarchy are close together helps to involve the co-workers in the decision-making. The team is a contributing factor for the co-workers to be able to influence both the care of the patients and their own working conditions. Working together with the focus on the patient is another factor that contributes to decreasing the hierarchy at the departments.

R: There's a very open atmosphere, there's no old-fashioned hierarchy

I: How come you don't have that in your department any more?

R: I don't know, because it hasn't really... I can't really remember that we've ever had anything like that in all the years I've worked there... There have sort of been very good doctors, but perhaps it's the fact that you've got to see the patient as a whole person at our department. (4)

#### Professionalism

The team spirit improves loyalty towards one's own occupational group as well as towards the entire working group. There are expressions of pride regarding both the common knowledge and ability in the present departments, as well as regarding professional competence:

Our head of department is very keen on us developing and learning, and really wants us to know our stuff. (3)

Belonging to a unique group (being specialists) promotes a professional attitude, which includes treating the patient correctly, as well as providing good treatment for the illness. According to the respondents there are many stressful situations. The staff support each other in dealing with these situations in a professional manner, together with workmates in collegial or professionally led counselling.

It can be really hard-going and then we have to help each other and sort of see the whole thing from a professional angle, but at the same time we've got to be allowed to have feelings too. (17)

## Discussion

In the results the picture of the departments might be considered too positive. Actually booth departments have gone through several difficulties such as mergers, and never-ending cuts in resources. Other difficulties have been conflicts about the philosophy of caring, as well as ongoing problems with organisational structural reconstruction. Consequently, these departments faced problems in the same way as others did. However, the intention of this study has been to concretise and increase understanding of what contributes to a healthy working life during a period of downsizing and restructuring. The results cannot provide definite answers concerning what are generally seen as determining factors for a healthy workplace, since there are certain given conditions for the two departments being studied with regard to the cooperation-oriented nature of their care and their small scale. Nevertheless, it is necessary to once again point out that these departments have faced the same demands for economic cuts and restructuring processes as the other departments at the hospital [38]. As a consequence of this, the proportion of their original staff has diminished from 1994, which has amongst other things meant an increasing trend to work hard. On the other hand, relatively fewer assistant nurses were laid off at the two selected departments compared with the rest of the hospital. In spite of this, the health of the personnel was more stable than that of other staff at other departments [5]. It is against this unstable organisational structure that the results are to be understood.

The informants comment on the value of belonging to a small department. According to Hodson [39], organisational size is a key determinant for job satisfaction and being able to feel pride in relation to one's work. Due to their size the departments in this study can continuously review, get to know each other and build up a sense of loyalty towards each other. In addition to this they have been able to develop a professional attitude with a clear professional identity. The opportunities for recurring personal interaction at small workplaces seem to increase the social capital at the workplace. A high degree of social capital, i.e. that the work team has a social network, norms and trust that facilitates cooperation, implies greater levels of satisfaction and quality of life at work [40,41]. The loyalty that is developed can also be explained in terms of social cohesion, i.e. a sense of belonging to a friendly, cohesive community [42]. Social contacts are thus a motivating factor at work and a determinant of health [22]. At the hospital in this study as a whole it emerged among other things that low group cohesion and low satisfaction with workmates were significantly associated with short-term sick leave during the period of organisational instability [5]. In other words, social capital and cohesion may have contributed to a stable health development at the departments in question, as it is known that work-related social support contributes to lower levels of work stressors [43]. Another contributing factor to the sense of cohesion may be that those who do not fit into the small department leave the organisation. A recently published study of long-term sick leave among women found that there was a greater risk of women tumbling out of the system in big and hierarchically organised workplaces [44]. From that perspective the small departments studied might be seen as protectors from long-term sick leave.

Both departments are highly specialised, and the staff are united by the clear common goal of providing the patients with care of high quality. Departmental goals are not enough in themselves; they must permeate the work of the department. According to Bass and Avolio [24], the ability of managers to empower their employees contributes to developing a climate where everyone works together to reach the common goals. The managers in this study, both at department level and ward level, appear to have the ability to get their co-workers to stretch their limits. Being given the opportunity to stretch yourself at work is considered stimulating. The managers seem to empower the co-workers to take responsibility for their work. Such an empowering leadership style [23] makes it easier for the employees to experience their competence and power to fulfil common goals [25]. The employees are given a more active role from their managers and thereby an opportunity to expand their decision latitude [13,14]. An empowering leadership style contributes to a more equal manager – employee relation. However, it is easy to exceed the limit for what is a reasonable workload when, as in this study, co-workers are willing and interested in their work, and prepared to take on extra tasks. There is a risk that important and stimulating work tasks become so motivating that they lead to illness instead of healthy motivation [2]. The managers in this study are aware of the danger of over-stimulating, but state that they may nevertheless fail to pay attention to the limits of individual co-workers' capacity.

In order to live up to the ambition of providing patients with specialised care of high quality, professional knowledge and skills are required, as well as well-developed cooperation, where everyone's competences are valuable contributions. The heads of department give their co-workers the opportunity to share the responsibility so that they can develop their competence. In this way the work environment can be characterised as empowerment-oriented, which can develop efficiency and job satisfaction at the workplace [26]. At a small and furthermore reduced work unit, the value and usefulness of each co-worker's efforts appear to be even more obvious.

Learning has high status at the departments. The management has realised the importance of competence development and, despite cuts, invests in integrating learning in the everyday work. Learning in everyday work is a resource that contributes to increasing the co-workers' sense of manageability [11]. In this way the individual is given the opportunity of having control over situations and his/her actions [12]. A learning organisation is also a positive determinant for increasing work motivation and making the work challenging as well as comprehensible and meaningful [11,12]. The results from the departments in this study are reminiscent of those that appeared in the interview studies conducted at the same hospital [2-4], where the female informants expressed a strong desire to work towards the same goal in a cooperating and learning team. For them this highly valued request was not a reality; however, this was the case for the two health-promoting departments. The competence development that the two departments invest in is directed towards the core activities of their respective departments. In order to bring about effective everyday learning there must be an opportunity for dialogue [45]. The management at the departments makes a conscious effort to provide opportunities for dialogue between individuals, within and between groups, and between different levels in the hierarchy. In this way they appear to have managed to break the hierarchical structures. At the same time as the teamwork has been developed, they have managed both to create confidence in each other and to retain the authority of the professional groups without creating jealously guarded territory. A possible interpretation of the result of this study is that the communication between the hierarchies is both top-down and bottom-up, which means that the management appears to have broken the communication pattern which the head nurses in Nilsson's study considered to be mostly top-down [46].

The style of management at the departments can be described as transformational, since the management strives to inspire and motivate co-workers to learn and develop. Intellectual stimulation as one aspect of transformational leadership deals with motivating employees to tax their resources to be innovative and creative, both in developing work tasks and solving problems [23]. The leadership style used in the departments leads in turn to the important components active problem-solving, support for coping, good communication and learning organisation. The managers are prepared to give their co-workers support and recognition. Personal recognition [47] could also define transformational style. In this respect the small size of the studied department could be an advantage, as managers and co-workers have many opportunities to communicate. By applying 'management by walking around' they could more easily pay attention to the individual employee [23].

When the organisation is in the middle of an unstable period, as is the case here, with increasing and conflicting work demands and less time to plan work, the co-workers' need for recognition, will probably be even more apparent. Recognition through feedback from the management can serve as an energy-giving activity [2-4] and thus a health promotion determinant. During the adjustment and restructuring phases, there has been an obvious trend that the personnel have worked harder and harder with a risk of overload [5]. At the present departments there is an awareness of these risks. There is a conscious managerial responsibility to keep a watchful eye on overload and to provide opportunity for recovery and time for reflection. Within this there is also the importance of integrating a certain measure of social activities in order to strengthen the group feeling.

There is a balancing act between giving co-workers responsibility and relieving them of it, as the management of the departments has done in a delicate way. Allowing co-workers to take responsibility for their work and their own development is good for productivity and efficiency [23], for work satisfaction [26,30] for decision-making [46] and as stress prevention [32]. The positive effects of being given, and taking, responsibility must be weighed against the risk of overloading the co-workers. In this balancing act the management uses empowerment strategies to delegate responsibility for carrying out the work parallel to strategies for prioritising work tasks. The support, which the co-worker perceives, helps him/her to gain control over the work, and this may have contributed to the health trend being stable at the departments [19,21].

## Conclusion

Taken together the core determinants seem to be work-pride and confidence. There is a pride in what is achieved in the departments; individually and together. This pride also includes professional pride, common knowledge and skills, as well as belonging to a certain department. Confidence is based on the strong support that exists at the workplace from managers and co-workers; everyone is there for the patients and for each other. The specific tasks of the departments contribute to the sense of belonging. The confidence in and respect for each other is strengthened when the co-workers cooperate with each other, and when there is collective responsibility for prioritising and for the development of knowledge and skills. In addition to this, the fact that the value of all personnel categories is emphasised by the managers, not only in words but also in action, appears to contribute to the expressed confidence. Our core determinants augment the well-established concepts manageability, comprehensiveness and meaningfulness. The core determinants interpreted in the themes organisational conditions, approaches and climate seem to be favourable conditions, functioning as a buffer against general negative effects of downsizing observed elsewhere in the hospital, and in the literature.

## Competing interests

The author(s) declare that they have no competing interests.

## Authors' contributions

KN: Study design, data collection, data analysis, writing the manuscript

AH: Study design, co-analyser, participating in writing the manuscript

ILP: Participating in writing the manuscript

TT: Contributed with discussions of consistence in the analysis

## Pre-publication history

The pre-publication history for this paper can be accessed here:


